# ELTD1 facilitates glioma proliferation, migration and invasion by activating JAK/STAT3/HIF-1α signaling axis

**DOI:** 10.1038/s41598-019-50375-x

**Published:** 2019-09-25

**Authors:** Junjun Li, Jianying Shen, Zhen Wang, Hao Xu, Qiangping Wang, Songshan Chai, Peng Fu, Tao Huang, Omarkhalil Anas, Hongyang Zhao, Jinsong Li, Nanxiang Xiong

**Affiliations:** 1 Department of Neurosurgery, Union Hospital, Tongji Medical College, Huazhong University of Science and Technology, Jiefang Street, Wuhan, 430022 P.R. China; 2 Department of Neurosurgery, Tongji Hospital, Tongji Medical College, Huazhong University of Science and Technology, Jiefang Street, Wuhan, 430030 P.R. China; 3 Section of Histology and Embryology, Department of Anatomy, Tongji Medical College, Huazhong University of Science and Technology, Hangkong Road, Wuhan, 430022 P.R. China; 4 Department of Thoracic surgery, Union Hospital, Tongji Medical College, Huazhong University of Science and Technology, Jiefang Street, Wuhan, 430022 P.R. China

**Keywords:** CNS cancer, Cell growth, Cell migration

## Abstract

The upregulation of ELTD1 ([epidermal growth factor (EGF), latrophilin and seven transmembrane domain-containing 1] on chromosome 1) in tumor cells has been reported in several types of cancer and correlates with poor cancer prognosis. However, the role of ELTD1 in glioma progression remains unknown. In this study, we examined ELTD1 expression levels in human glioma cell lines and in sixteen human gliomas of different grades. The molecular effects of ELTD1 in glioma cells were measured using quantitative polymerase chain reaction (qRT-PCR), Western blotting, Cell proliferation assays, Matrigel migration and invasion assays and brain orthotopic xenografts. We found that high expression levels of ELTD1 were positively associated with cancer progression and poor prognosis in human glioma. Mechanistically, ELTD1 activated the JAK/STAT3/HIF-1α signaling axis and p-STAT3 bound with HIF-1α. Taken together, our data provide a plausible mechanism for ELTD1-modulated glioma progression and suggest that ELTD1 may represent a potential therapeutic target in the prevention and therapy of glioma.

## Introduction

Glioma is the most prevalent tumor of CNS (central nervous system), and both the morbidity and mortality rates for gliomas have increased in recent years^1,2^. Surgical intervention, chemotherapy and radiotherapy are the most common forms of therapy for glioma, but the patient prognosis is still not optimal due to tumor multifocal development and recurrence characteristics^3,4^. Thus, elucidating intrinsic particular mechanism of its invasive properties for glioma is pivotal for the identification of novel targets.

ELTD1 is a secretory family from G protein coupled receptor^5^. ELTD1 includes a domain similar to EGF, a short cytoplasmic tail and a seven-trans-membrane domain^5^. It is reported for the first time that the function of ELTD1 is in postnatal cardiomyocytes and rat fetuses^5^. In recent years, the role of ELTD1 has also been reported in rheumatoid arthritis^6^. In addition, ELTD1 has also been reported to be involved in the sensitivity of anesthetics^7,8^, subcutaneous fat thickness^9^ and tick burden in cattle^10^. In terms of tumor, it has been used as a microvascular endothelial marker^11^. Towner *et al*. reported that ELTD1 is a marker for glioma^12^. Ziegler J *et al*. had reported that it is an effective anti-angiogenic target for gliomas^13^. Besides, Serban F *et al*. reported that the silencing of ELTD1 can induce glioblastoma cell death^14^ and it is a novel angiogenesis marker^15^. Tower RA *et al*. had also reported that ELTD1 might be a potential target for rodent gliomas^16^. In addition, Dai S *et al*. had also proved that miR-139-5p inhibits tumor progression via targeting ELTD1^17^.

Signal transducer or STAT3 is activated by JAK and they are very critical for cells growth and differentiation as a regulatory transcription factor^18^. In tumor cells, STAT3 has been shown to regulate HIF-1α^19^. STAT3 can inhibit the degradation pathway of HIF-1α^20^. A wealth of evidence proves that high expression levels of HIF-1α in solid tumors and during tumor growth is significantly limited after knocking it out, which indicates that it exerts a vital role in cancer development^21–23^.

Here, we elucidated the mechanism of ELTD1 in glioma. This study showed that ELTD1 is often over-expressed in glioma. Besides, *in vitro* and *in vivo* experiments have shown that ELTD1 exerts a vital role in facilitating glioma cells proliferation, migration, and invasion. Our results also indicated that the JAK/STAT3/HIF-1α signaling may mediate this process.

## Results

### ELTD1 is often overexpressed and correlates with poor prognosis in glioma

**Figure 1:**
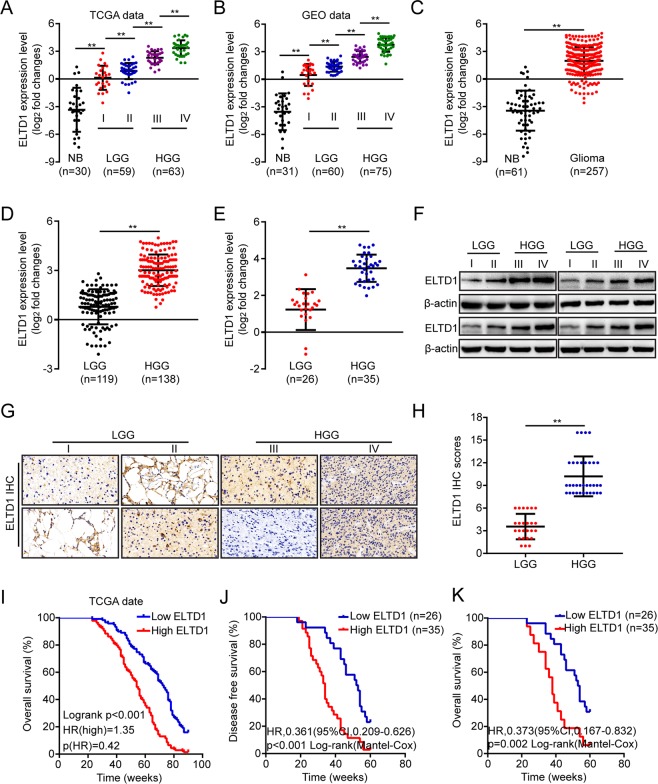
ELTD1 is often overexpressed and correlates with poor prognosis in glioma. (**A**) The expression of ELTD1 in normal brain tissues (NB) and glioma tissues (LGG: Low-grade glioma, HGG: High-grade tissue) obtained from the TCGA database. The nonparametric Mann–Whitney U-test was used. (**B**) The expression of ELTD1 in normal brain tissues (NB) and glioma tissues obtained from the GEO database (GSE16011, GPL8542, Affymetrix GeneChip Human Genome U133 Plus 2.0 Array, Hs133P_Hs_ENTREZG.cdf). The nonparametric Mann–Whitney U-test was used. (**C**) The expression of ELTD1 in normal brain tissues (NB) and glioma tissues obtained from the TCGA and GEO database. The nonparametric Mann–Whitney U-test was used. (**D**) The expression of ELTD1 in LGG and HGG obtained from the TCGA and GEO database. The nonparametric Mann–Whitney U-test was used. (**E**) Relative ELTD1 expression levels measured by RT-PCR in 26 LGG and 35 HGG. (**F**) Sixteen glioma tissues were assessed for ELTD1 expression by Western blotting, including four grade I, four grade II, four grade III and four grade IV. (**G**,**H**) Representative images (**G**) and scores (**H**) of the IHC of ELTD1 expression (blue) in the paraffin-embedded different grade glioma. (**I**–**K**) Kaplan–Meier curves for OS (**I**,**K**) and DFS (**J**) of glioma patients with low vs. high expression of ELTD1. The median ELTD1 expression was used as the cutoff value. Statistical significance was assessed using two-tailed Student’s t test (**C**–**E**, **H**–**K**) and one-way ANOVA followed by Dunnett’s tests for multiple comparisons (**A**,**B**). Scale bars: 50 μm. **p < 0.01 and ***p < 0.001.

Sixty-one normal brain tissue and 257 glioma (49 gradeI, 70 gradeII, 65 grade III and 73 grade IV) tissues were obtained from TCGA and GEO. We found that the ELTD1 expression levels in the tumors were significantly higher than those in normal brains, especially in high-grade glioma (III + IV), by analyzing the bioinformatics data (Fig. 1A–D, P < 0. 01). The ELTD1 expression levels in gliomas of various grades were measured by RT-PCR. We found that the ELTD1 expression levels were consistently upregulated in high-grade gliomas (III + IV) (Fig. 1E, P < 0. 01). Sixteen glioma tissues were used to measure the ELTD1 expression by Western blotting, including four grade I, four grade II, four grade III and four grade IV tissue samples. Western blotting analyses showed that ELTD1 was often overexpressed in high-grade gliomas (III + IV) (Fig. 1F). Then immunohistochemical (IHC) analysis was employed to detect the expression level of ELTD1 in 61 glioma samples. As shown in Fig. 1G, the IHC staining intensity of ELTD1 was notably different in different grades of glioma, and a quantification analyses further indicated that ELTD1 protein expression was significantly elevated in high-grade cancer (III + IV) (Fig. 1H, P < 0.01). The correlation between ELTD1 and clinico-pathological features of 61 glioma samples was statistically analyzed. The results showed that ELTD1 overexpression was significantly correlated with the Karnofsky Performance Scale (KPS) score (P = 0.003) and tumor recurrence (P = 0.03, Table 1). This result was consistent with the KPS as an independent predictor for survival^24^. As shown in Table 2, using Univariate Cox regression analyses, ELTD1 overexpression was related to a notably increased risk of tumor recurrence in glioma patients (P = 0.03) compared to the risk of recurrence in patients with low expression levels (Table 2). Multivariate Cox regression analyses demonstrated that ELTD1 could predict poor prognosis when ELTD1 expression (*P* = 0.038), tumor grade (*P* = 0.021) and tumor recurrence (*P* = 0.042) were included in the analysis (Table 2). These results demonstrate a significant correlation between ELTD1 expression and prognosis. Furthermore, a Kaplan-Meier analysis showed that ELTD1 overexpression was significantly correlated with poorer disease-free survival (DFS) and overall survival (OS) rates in glioma patients (Fig. 1I–K). The median ELTD1 expression was used as the cutoff value (SI = 8). Samples with SI ≥8 had high expression, and samples with SI <8 had low expression. Follow-up time was 90 weeks (I) and 60 weeks (J–K) respectively. Overall survival (OS) is defined as the time from the start of randomization to the death of any cause. Disease-free survival (DFS) is defined as the time from the start of randomization to the recurrence of disease for any reason. The X values are time. The Y values are entered into two columns that define the treatment groups. The Y value is “1” when the subject died at the specified time, and “0” when the subject’s data was censored at that time. Then we input the corresponding X and Y into GraphPad Prism 7 to generate the corresponding graph and perform corresponding statistical analysis.

**Table 1:** Association of ELTD1 expression with clinicopathological characteristics in human glioma.

Features	No.	ELTD1		P-value
		Low	High	
**Age, years**				
<40	35	16	19	0.57
> = 40	26	10	16	
**Gender**				
Male	34	18	16	0.07
Female	27	8	19	
**Tumor size, cm**				
<3	37	22	15	0.001
> = 3	24	4	20	
**Tumor location**				
Supratentorial	40	17	23	0.98
Subtentorial	21	9	12	
**Karnofsky performance scale**				
<90	38*	11	27*	0.003
> = 90	22	15	7	
WHO grade				
Low-grade (I + II)	26	16	10	0.01
High-grade (III + IV)	35	10	25	
**Tumor recurrence**				
No	40	21	19	0.03
Yes	21	5	16	

*Partial data not available; statistics based on available data.

**Table 2:** Univariate and Multivariate analyses of various prognostic parameters in patients with glioma using Cox-regression analysis.

	Univariate analysis			Multivariate analysis		
	p value	Hazard Ratio	95% confidence interval	p value	Hazard Ratio	95% confidence interval
ELTD1	0.035	1.243	1.135–2.254	0.038	1.268	1.192–2.178
Tumor size, cm	0.001	1.563	1.313–3.794	0.013	1.412	1.265–3.417
Karnofsky performance scale	0.003	1.475	1.261–3.636	0.025	1.398	1.235–2.637
WHO grade	0.01	1.326	1.187–2.243	0.021	1.287	1.176–2.459
Tumor recurrence	0.03	1.276	1.236–2.413	0.042	1.215	1.125–2.437

### ELTD1 overexpression promotes glioma cell proliferation, migration and invasion

**Figure 2:**
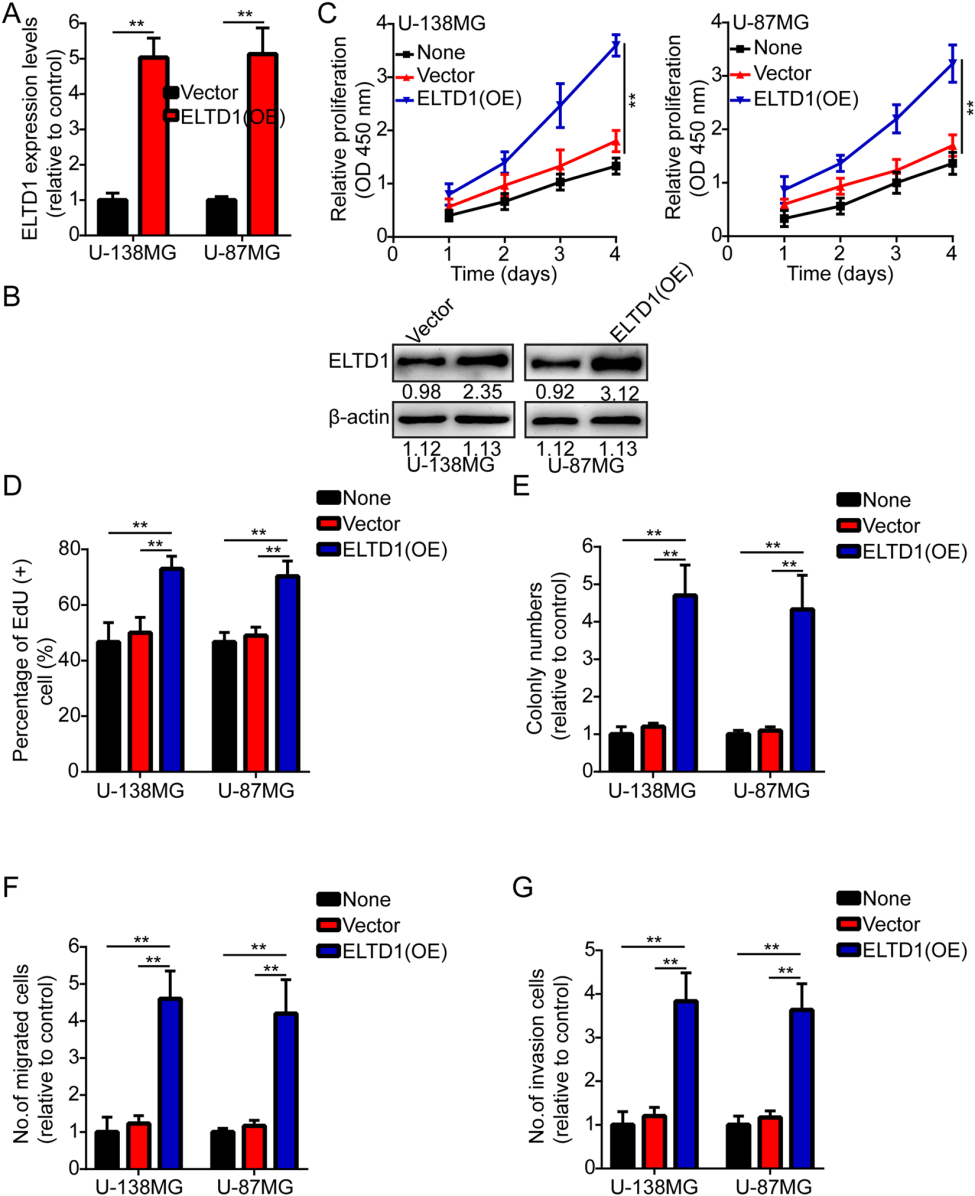
ELTD1 overexpression promotes glioma cell proliferation, migration and invasion. (**A**,**B**) The overexpression efficiency against ELTD1 was verified by RT-PCR and Western blotting in U-87MG and U-138MG cells. (**C**) Growth curves between None, Vector and ELTD1 (OE) by CCK-8 assay. The results are shown as the Mean ± Standard Deviation (SD) of five independent experiments. (**D,E**) Representative images (left panels) and histogram quantification (right panels) of the EdU (**D**) and colony formation assay (**E**) with U-87MG and U-138MG cells. (**F,G**) Representative images (left panels) and histogram quantification (right panels) of the Transwell migration (**F**) and invasion assays (**G**) with U-87MG and U-138MG cells. Statistical significance was assessed using two-tailed Student’s t test (**A**) and one-way ANOVA followed by Dunnett’s tests for multiple comparisons (**C**–**G**). Scale bars: 50 μm. **p < 0.01.

First, we measured ELTD1 expression levels by RT-PCR and Western blotting in two human brain gliocyte cell lines and six human brain glioma cell lines. The results showed that ELTD1 was overexpressed in the glioma cell line (H4, A-172, U-138MG, LN-18, U-87MG and U-87MG) compared with its expression in the human brain gliocyte cell line (HA and HM) (Supplement 4). The role of ELTD1 in tumor progression was researched because its overexpression was associated with tumor size and recurrence. U-87MG and U-138MG cells were stably transfected with lentiviruses containing ELTD1, and the overexpression efficiency was verified by RT-PCR and Western blotting (Fig. 2A,B). Then, we employed CCK-8, EdU, colony formation and Transwell migration and invasion assays to identify the effect of ELTD1 on the proliferation, migration and invasion of cancer cells. The results indicated that ELTD1 overexpression notably promoted cancer cell proliferation, migration and invasion (Fig. 2C–G, Supplement 1).

### ELTD1 knockdown suppresses glioma cell proliferation, migration and invasion

**Figure 3:**
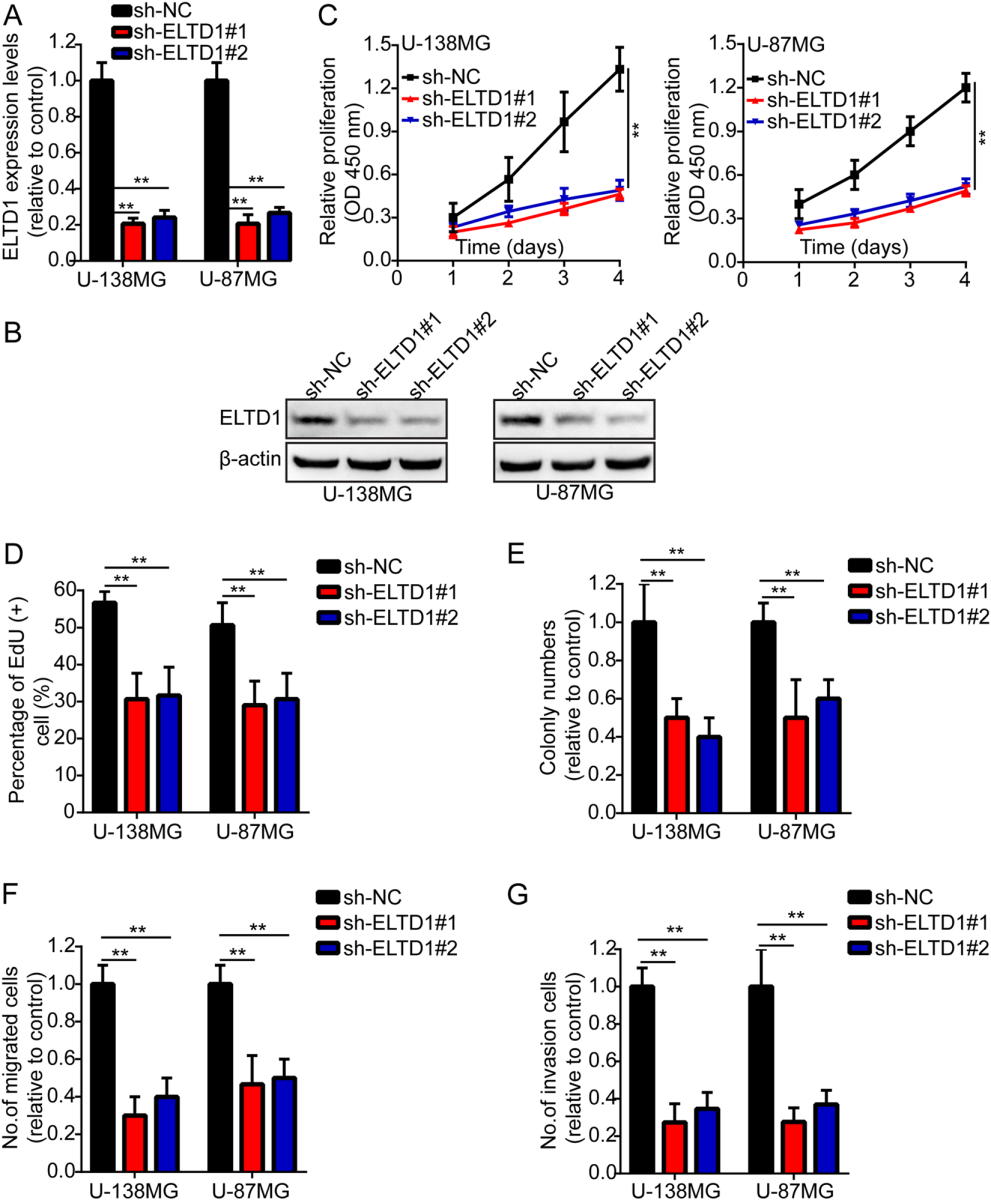
ELTD1 knockdown suppresses glioma cell proliferation, migration and invasion. (**A**,**B**) The knockdown efficiency against ELTD1 was verified by RT-PCR and Western blotting in U-87MG and U-138MG cells. (**C**) Growth curves between sh-NC, sh-ELTD#1 and sh-ELTD#2 by CCK-8 assay. The results are shown as the Mean ± Standard Deviation (SD) of five independent experiments. (**D**,**E**) Representative images (left panels) and histogram quantification (right panels) of the EdU (**D**) and colony formation assay (**E**) with U-87MG and U-138MG cells. (**F**,**G**) Representative images (left panels) and histogram quantification (right panels) of the Transwell migration (**F**) and invasion assays (**G**) with U-87MG and U-138MG cells. Statistical significance was assessed using one-way ANOVA followed by Dunnett’s tests for multiple comparisons. Scale bars: 50 μm. **p < 0.01.

Then we knocked down ELTD1 with short hairpin RNAs in U-87MG and U-138MG cells. The knockdown efficiency was also verified by RT-PCR and Western blotting (Fig. 3A,B). Subsequently, we used the above experiments to measure the effect of ELTD1 on the proliferation, migration and invasion of cancer cells. The results showed that ELTD1 knockdown notably suppressed cancer cell proliferation, migration and invasion (Fig. 3C–G, Supplement 2).

### ELTD1 regulates glioma cell proliferation, migration and invasion via the JAK/STAT3 signaling pathway

**Figure 4:**
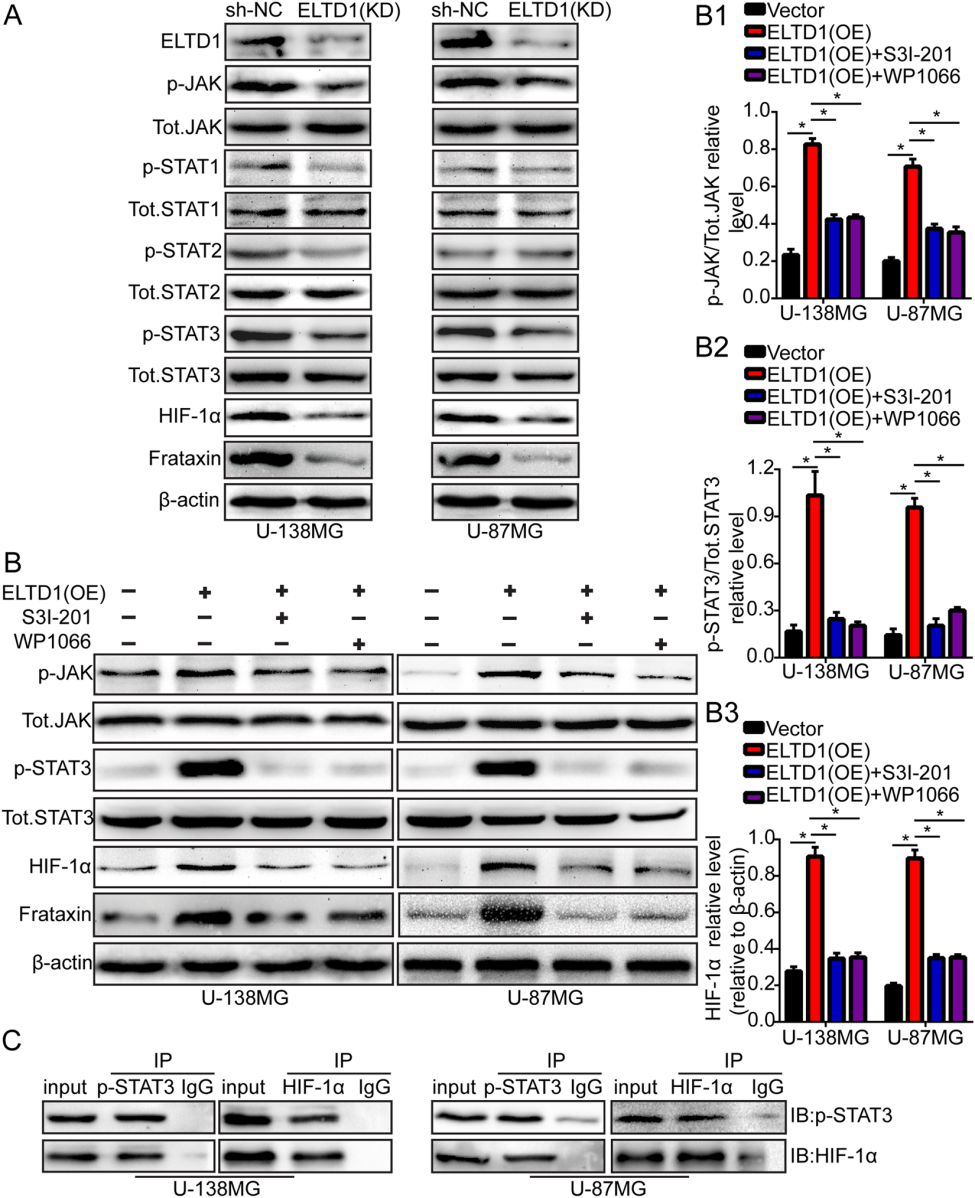
ELTD1 regulates glioma cell proliferation, migration and invasion via the JAK/STAT3 signaling pathway. (**A**) ELTD1 knockdown decreased the protein level of p-JAK, p-STAT3, HIF-1α and Frataxin. Other proteins remain unchanged. (**B**) U-87MG and U-138MG cells transfected with ELTD1 plasmid and co-cultured with JAK/STAT signaling inhibitors were reaped, and the lysates were immuneblotted for p-JAK, Tot.JAK, p-STAT3, Tot.STAT3, HIF-1α, Frataxin and β-actin. The histogram quantification (right panels) of p-JAK/ Tot.JAK (**B1**), p-STAT3/ Tot.STAT3 (**B2**) and HIF-1α/ β-actin (**B3**). (**C**) Co-IP experiment indicated that p-STAT3 could bind with HIF-1α. Statistical significance was assessed using one-way ANOVA followed by Dunnett’s tests for multiple comparisons. *p < 0.05.

To identify the potential mechanism of ELTD1-regulated glioma cell progression, the Cignal Finder Cancer 10-Pathway Reporter Kit was employed to screen for signaling pathways that were possibly involved in this process. The final results indicated that the JAK/STAT signaling pathway was notably inhibited, but the other signaling pathways were not markedly affected by ELTD1 knockdown in U-87MG and U-138MG cells (Supplement 5). To clarify this result, the dual luciferase reporter assay was employed in U-87MG and U-138MG cells. Consistently, the results indicated that ELTD1 knockdown could inhibit the JAK/STAT signaling pathway, which was consistent with the signaling pathway screening outcome (Supplement 5). Then, we knocked down ELTD1 with short hairpin RNAs in U-87MG and U-138MG glioma cells and found that the protein levels of p-JAK, p-STAT3, HIF-1α, and Frataxin notably decreased compared with the levels in the control group. Moreover, the other protein levels were unchanged (Fig. 4A). To validate the results, we cocultured ELTD1-overexpressing cells with two chemical inhibitors of the JAK/STAT signaling pathway, WP1066 and S3I-201. Initially, their inhibition efficiency in U-87MG and U-138MG cells was shown by p-JAK (Fig. 4B, B1). The p-STAT3 protein expression level after ELTD1 overexpression in U-87MG and U-138MG cells was attenuated by WP1066 and S3I-201 (Fig. 4B, B2). In addition, the HIF-1α and Frataxin proteins expression levels after ELTD1 overexpression in U-87MG and U-138MG cells were also attenuated by WP1066 and S3I-201 (Fig. 4B, B3). The Co-IP experiments indicated that p-STAT3 could bind with HIF-1α (Fig. 4C, Supplement 7). All the results strongly indicated that ELTD1 overexpression promoted HIF-1α protein expression through the JAK/STAT3 signaling pathway.

### HIF-1α is involved in ELTD1-regulated glioma cell proliferation, migration and invasion

**Figure 5:**
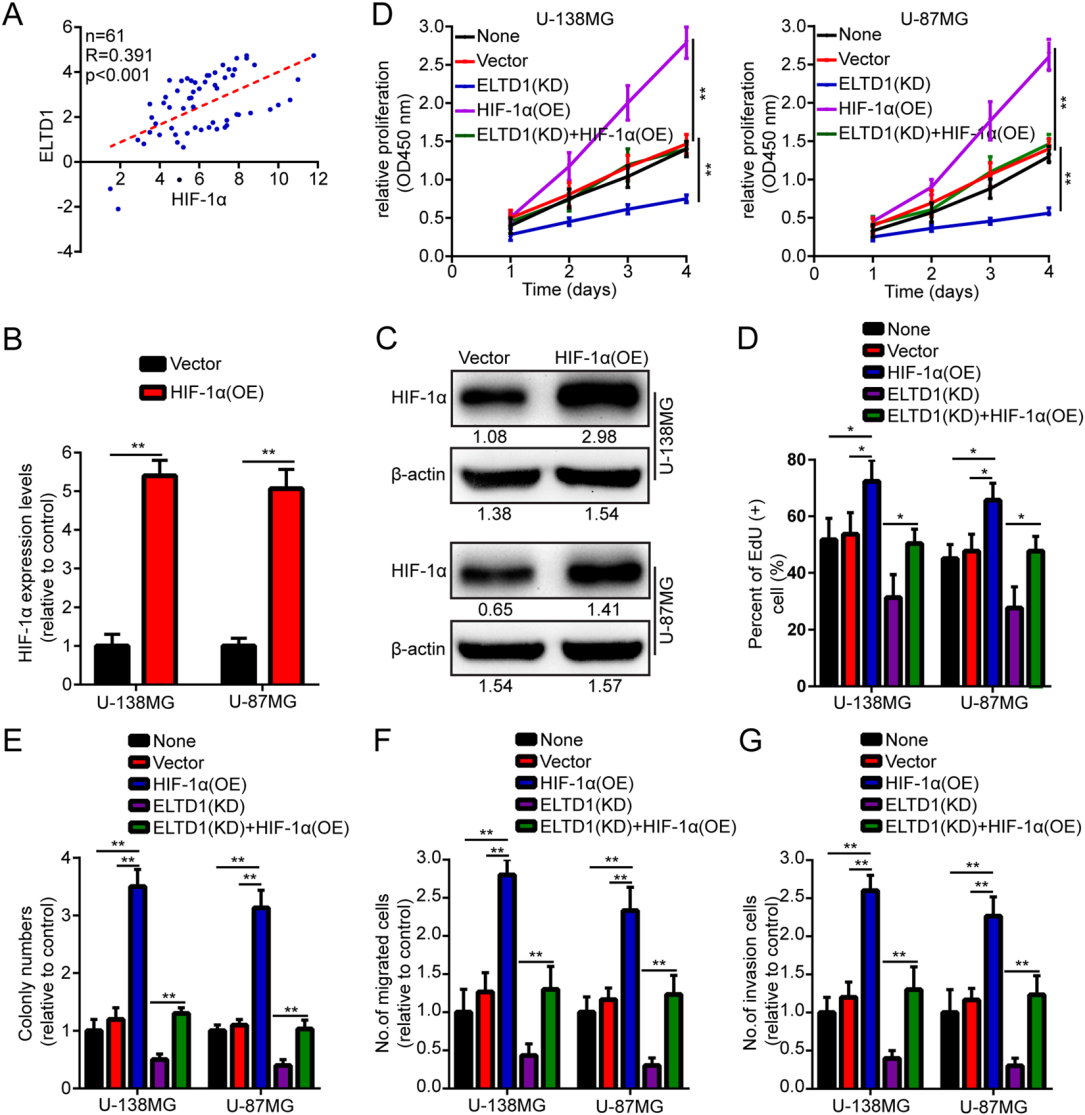
HIF-1α is involved in ELTD1-regulated glioma cell proliferation, migration and invasion. (**A**) Correlation analysis of ELTD1 and HIF-1α mRNA expression by RT-PCR. (**B**,**C**) The overexpression efficiency against HIF-1α was verified by RT-PCR and Western blotting in U-87MG and U-138MG cells. (**D**) Growth curves between None, Vector, ELTD1 (KD), HIF-1α (OE) and ELTD1 (KD) + HIF-1α (OE) by CCK-8 assay. The results are shown as the Mean ± Standard Deviation (SD) of five independent experiments. (**E**,**F**) Representative images (left panels) and histogram quantification (right panels) of the EdU (**E**) and colony formation assay (**F**) with U-87MG and U-138MG cells. (**G**,**H**) Representative images (left panels) and histogram quantification (right panels) of the Transwell migration (**G**) and invasion assays (**H**) with U-87MG and U-138MG cells. All the results indicated that HIF-1α overexpression abrogated the effects of ELTD1 knockdown on inhibiting U-87MG and U-138MG cell proliferation (**D**,**E**), colony formation (**F**), migration (**G**) and invasion (**H**). Statistical significance was assessed using two-tailed Student’s t test (**A**,**B**) and one-way ANOVA followed by Dunnett’s tests for multiple comparisons (**D**–**H**). Scale bars: 50 μm. *p < 0.05, **p < 0.01 and ***p < 0.001.

It has been reported that HIF-1α is highly expressed in parenchymal tumors and that tumor growth is significantly limited after HIF-1α is knocked down^25–27^. Accumulating evidence indicates that HIF-1α protects hypoxic cells and plays an important role in regulating tumor formation^28–31^. Therefore, we hypothesized that ELTD1 might upregulate HIF-1α expression, thereby inducing tumor cell growth, migration and invasion. First, we measured the expression levels of HIF-1α and ELTD1 in 61 glioma samples using RT-PCR to identify the correlation between ELTD1 and HIF-1α. The data indicated that they are positively correlated (Fig. 5A). Next, we overexpressed HIF-1α in U-87MG and U-138MG cells to verify whether HIF-1α is involved in ELTD1-regulated glioma cell proliferation (Fig. 5B,C). As expected, HIF-1α overexpression abrogated the effects of ELTD1 knockdown on inhibiting U-87MG and U-138MG cell proliferation (Fig. 5D,E), colony formation (Fig. 5F), migration (Fig. 5G) and invasion (Fig. 5H) (Supplement 3). Taken together, our results indicate that ELTD1 promotes the proliferation, migration and invasion of glioma cells by regulating HIF-1α protein expression.

### ELTD1 knockdown inhibits the growth of the brain orthotopic tumor model *in vivo*

**Figure 6:**
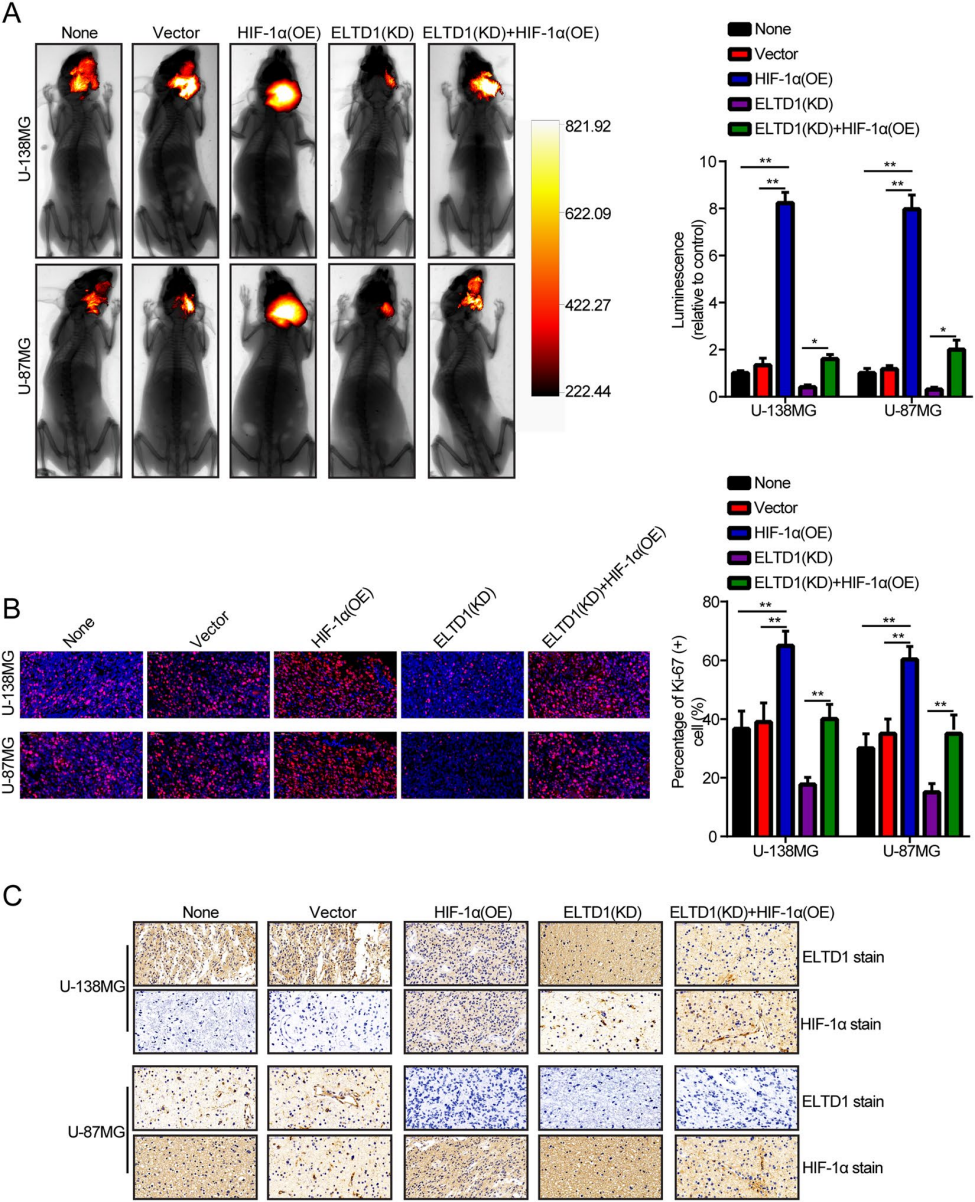
ELTD1 knockdown inhibits the growth of the brain orthotopic tumor model *in vivo*. (**A**) Representative images of the brain orthotopic tumor by glioma cells injected into the brain parenchyma of nude mice (left panels) and histogram analysis of luminescence representing the size of the tumors measured on day 28 (right panels). n = 5. (**B**) Representative images (left panels) of IF staining with Ki-67 and histogram analysis of the percentage of Ki-67 ( + ) cells (right panels). (**C**) Representative images of H&E staining, IHC staining with ELTD1 and HIF-1α. Statistical significance was assessed using one-way ANOVA followed by Dunnett’s tests for multiple comparisons. Scale bars: 50 μm. *p < 0.05 and **p < 0.01.

To elucidate the function of ELTD1 in glioma, nude mice orthotopic tumor model was employed. The U-87MG/Luc and U-138MG/Luc glioma cells over-expressing HIF-1α or knockdown ELTD1 were injected into the animal brain parenchyma (n = 5). Four weeks after injection, the size of the tumors in the nude mice was quantified by measuring the luminescence intensity using animal living imager (BRUKER, USA). All results indicated that ELTD1 knockdown could inhibit tumor growth and that HIF-1α overexpression could abrogate the ELTD1 knockdown-mediated inhibition of tumor growth (Fig. 6A). Immunofluorescence of Ki-67 indicated that ELTD1 knockdown inhibited its expression relative to the control groups. Conversely, HIF-1α overexpression abrogated the ELTD1 knockdown-mediated inhibition of Ki-67 (Fig. 6B). In addition, Immunohistochemistry presented the protein expression levels of ELTD1 or HIF-1α in different groups (Fig. 6C). Collectively, these data suggested that ELTD1 knockdown could inhibit tumor growth and that HIF-1α overexpression could abrogate the effect *in vivo*.

## Discussion

Glioma accounts for approximately half diagnosed primary CNS cancer. Glioblastomas (GBM) are the most common malignant type among gliomas, and are related to short overall survival time because the OS upon diagnosis is approximately 15 months^32–34^. Therefore, it is very urgent to identify new biomarkers and targets for anticancer treatment.

Herein, the data obtained from TCGA and GEO demonstrated that the ELTD1 mRNA expression levels in the tumors, especially in HGG (III + IV), were notably up than them in normal brains. Then, we measured ELTD1 expression levels in sixteen glioma samples by RT-PCR and Western blotting. These results indicated that ELTD1 is often upregulated in HGG (III + IV). Then, we performed a correlation analysis between the mRNA expression levels of ELTD1 and clinical parameters, which suggested ELTD1 as a potential new clinical prognostic index. Rheal A. Towner *et al*. reported that ELTD1 could act as a potential new biomarker for glioma^12^ and Ziegler J *et al*. also reported that ELTD1 may be a target for effective anticancer therapy for glioma^13^. Besides, Serban F *et al*. reported that the silencing of ELTD1 can induce glioblastoma cell death^14^ and it is a novel angiogenesis marker^15^. However, the exact mechanism of ELTD1 anticancer therapy has not been elucidated. Here, our experimental results showed that ELTD1 could promote the progression of glioma by promoting glioma cells growth. Through the functional experiments of cells, we proved that ELTD1 promotes glioma cells growth, migration and invasion. ELTD1 knockdown notably reduced glioma cells growth *in vitro* and *in vivo*. Conversely, ELTD1 over-expression promoted glioma cells growth *in vitro*.

Subsequently, screening of signal pathways through related experiments, our experimental results showed that ELTD1 could affect JAK/STAT signaling pathway. This signaling pathway exerts very vital roles in cancer growth or metastasis^35–38^. STAT3 has been reported to play a crucial role in cells survival, proliferation, and differentiation^18^. STAT3 is an original regulatory molecule of HIF-1α and can suppress its degradation^39^. The following results showed that ELTD1 knockdown inhibited JAK/STAT reporter activity. In short, ELTD1 promotes phosphorylation of JAK protein, phosphorylated JAK protein promotes phosphorylation of STAT3 protein, and phosphorylated STAT3 protein promotes HIF-1α protein expression levels, suggesting that it is the downstream effector of ELTD1. Importantly, the Co-IP experiment revealed that p-STAT3 could bind with HIF-1α. Indeed, the Supplementary results showed that down-regulation of JAK/STAT was observed in ELTD1-silenced cells and the expression of p-JAK, p-STAT1–3, and HIF-1α was also suppressed in ELTD1-silenced cells. However, we detected the change of downstream molecules by knocking down ELTD1 and using Western blot. Our data show that the change of p-STAT3 is the most obvious. Due to a large number of reports, the downstream effective molecule of STAT3 is HIF-1α. Therefore, we speculated and verified the signal axis of ELTD1-JAK/STAT3-HIF-1α. There is no report on direct combination between them in the literature, so we used CO-IP experiments to verify the interaction of two proteins (F4 C). Of course, we could not rule out the role of STAT1 in the present study. As we all know, numerous reports have reported that STAT1 can also regulate HIF-1α^40,41^ and there may be many proteins downstream of ELTD1. In this study, we have only selected and validated one of the most important downstream effective proteins with obvious changes (STAT3).

In a word, this research elucidated the function of ELTD1 in glioma and found that it is often highly expressed in tumor tissues. ELTD1 promotes the progression of glioma through the JAK/STAT3/HIF-1α axis. Targeted inhibition of this signal axis may have a certain effect on cancer treatment.

## Materials and Methods

### Bioinformatics database

Related data were downloaded from the TCGA (The Cancer Genome Atlas)^42,43^. See Supplementary Materials for details.

### Cell lines and reagents

See Supplementary Materials for details.

### Patients and sample preparation

Sixty-one surgically resected glioma samples were collected between October 2016 and September 2018 at Union Hospital, Tongji Medical College, Huazhong University of Science and Technology. Ethical consent was approved by the ethics committee involving human subjects at Tongji Medical College, Huazhong University of Science and Technology (S360). Written informed consent was obtained from all patients before sample collection. All methods were performed in accordance with approved guidelines. Prior to glioma resection, no patient received radiotherapy or chemotherapy. All samples were immediately snap-frozen in liquid nitrogen and stored at liquid nitrogen until required. This research was performed in accordance with the Helsinki Declaration.

### Plasmid construction and transfection

More details can be found in the Supplementary Materials and Methods.

### Cell proliferation assays

Cell growth was assessed using CCK-8, EdU and Colony formation assays as described in the Supplementary Materials and Methods.

### Cell migration and invasion assays

Cell migration and invasion experiments were performed using the Transwell system (Corning, NY) based on the manufacturer’s instructions. More details can be found in the Supplementary Materials and Methods.

### Dual luciferase reporter assay

More details can be found in the Supplementary Materials and Methods.

### Cignal finder cancer 10-pathway reporter array

Pathway analyses were performed with the Cignal Finder Cancer 10-Pathway Reporter Array (QIAGEN, Germany) according to the manufacturer’s instructions. More details can be found in the Supplementary Materials and Methods.

### Western blotting

The process was performed as described previously. More details can be found in the Supplementary Materials and Methods.

### qRT-PCR

The protocol and primers used are provided in the Supplementary Materials and methods. Relative mRNA expression levels were normalized as described previously^44,45^.

### Co-IP (Coimmunoprecipitation)

Details are included in the Supplementary Materials and Methods.

### Immunofluorescence and immunohistochemical staining

Immunofluorescence (IF) staining was performed as described previously^46,47^. Immunohistochemistry and semiquantitative scoring techniques were performed as described previously^48^. More details are included in the Supplementary Materials and Methods.

### Brain orthotopic xenografts

All animal experiments were approved by the Institutional Animal Care and Use Committee of Tongji Medical College, Huazhong University of Science and Technology (S841). More details can be found in the Supplementary Materials and Methods.

### Statistical analyses

All data are presented as the Mean ± Standard Deviation (SD) from at least 3 independent experiments. The unpaired/paired Student’s t test was used to identify statistically significant data between two groups and one-way ANOVA followed by Dunnett’s multiple comparisons tests was used to identify statistically significant data between more than two groups. Overall survival (OS) and disease-free survival (DFS) were evaluated using Kaplan–Meier method and Multivariate survival analyses were performed using a Cox regression model. All statistical analyses were performed with using the GraphPad Prism version 7 (GraphPad Inc., La Jolla, CA, USA). P-values < 0.05 were considered statistically significant.

### Ethics approval and consent to participate

The use of human tissues was approved by the Human Research Committee of Huazhong University of Science and Technology (S360). Written informed consent was obtained from each patient. All animal experiments were performed according to the guidelines of care and use of laboratory animals and were approved by the Tongji Medical College Animal Experiments Committee (S841).

## Supplementary information

Supplementary Materials and methods

Language Certificate

## Data Availability

The datasets used during the present study are available from the corresponding author upon reasonable request.
