# Supplementary material for: ELTD1 facilitates glioma proliferation, migration and invasion by activating JAK/STAT3/HIF-1α signaling axis
Source: Sci Rep. 2019 Sep 25;9:13904. doi: 10.1038/s41598-019-50375-x (PMC6761139; doi:10.1038/s41598-019-50375-x)
Supplement: Supplementary file 1 — Supplementary Materials and methods [file 41598_2019_50375_MOESM1_ESM.docx]

# ELTD1 facilitates glioma proliferation, migration and invasion by activating JAK/STAT3/HIF-1α signaling axis

Junjun Li^1^, Jianying Shen^3^, Zhen Wang^2^, Hao Xu^1^, Qiangping Wang^1^, Songshan Chai^1^, Peng Fu^1^, Tao Huang^1^, Omarkhalil Anas^3^, Hongyang Zhao^1^, Jinsong Li^4^, Nanxiang Xiong^1^

**Materials and methods**

**Bioinformatics Database**

For the glioma expression microarray analysis, raw Affymetrix.CEL files were downloaded from the TCGA (National Cancer Institute (NCI) The Cancer Genome Atlas; www.cancergenome.nih.gov/), Rembrandt (NCI Repository for Molecular Brain Neoplasia Data; www.rembrandt.nci.nih.gov), and Erasmus (NCBI Gene Expression Omnibus; GEO Series GSE16011) databases; the corresponding clinical annotations of each file were also obtained. Then the raw Affymetrix.CEL files were processed using R language and Bioconductor using a custom CDF (custom chip definition files) with background correction, log transformation, and quantile normalization performed using the RMA (robust multiarray average) algorithm in R.

**Cell lines and reagents**

HM, HA, H4, A-172, U-251MG, LN-18 (Cellcook Biotech Co, Ltd, Guangzhou), U-138MG and U-87MG cells (glioblastoma of unknown origin) (American Type Culture Collection) were cultured in complete medium. All cell lines were authenticated by short tandem repeats (STR) analysis and confirmed to be mycoplasma negative every 3 months. [**S3I-201**](https://www.selleck.cn/products/S31-201.html) and [**WP1066**](https://www.selleck.cn/products/wp1066.html) were purchased from SelleckChem (Selleck.cn, Houston, Texas, USA). Anti-ELTD1 antibody (ab150489) and Anti-HIF-1α antibody (ab92498) were purchased from Abcam (USA); Anti-JAK (9945), Anti-p-JAK (66245), anti-STAT1 (14995), anti-p-STAT1 (Ser727) (8826); anti-STAT2 (72604), anti-p-STAT2 (Tyr690) (88410); anti-STAT3 (4904), anti-p-STAT3 (Tyr705) (9145) and anti-β-actin antibodies (4970) were bought from Cell Signaling Technology (Beverly, MA, USA).

**Plasmid construction and transfection**

To establish stable knockdown and overexpression cell lines, full-length shRNA sequences that specifically target ELTD1 or HIF-1α were cloned into vectors of pLKO.1-MSCV-Puro (with or without luciferase) or pcDNA3.0 (with or without luciferase) and were bidirectionally sequenced. Sequences of all shRNAs are provided in Supplemental Table 1. The construction of the plasmid and the packaging of the lentivirus were completed GenePharma by the company (GenePharma, Shanghai, China). Cells were infected by the lentivirus according to the manufacturer’s protocol and were selected by puromycin (Sigma-Aldrich, St Louis, MO, USA) for two weeks to obtain cell lines with stable expression. The empty vector pcDNA3.0 or pLKO.1 and scrambled siRNA were used as a negative control.

**Cell proliferation assays**

For the CCK-8 (Cell Counting Kit-8) assay, cells were counted and plated in complete culture medium at 4000/well in 96-well plates. After treatment with 10μl CCK-8 (Dojindo Laboratories, Japan), the absorbance at 450 nm was detected with using a microplate reader (Spectra Max M2 reader, Molecular Devices, USA) and each experiment was performed in triplicate.

**EdU proliferation assay**

Newly synthesized DNA after the indicated treatment in U-87MG and U-138MG cells was measured by EdU fluorescence staining based on the manufacturer’s protocol (Click-iT^®^ EdU Imaging Kits, Invitrogen). The cells, cultured in 96-well plates at a density of 5 × 10^4^ cells/well, were labeled with 10 μM EdU, incubated for 3 h, and then fixed for 20 min with 3.7% formaldehyde at room temperature. The fixative was removed, and the cells in each well were washed three times with 3% BSA in PBS. The BSA was removed, and the cells were permeabilized with 0.5% Triton X-100 (Sigma, San Francisco, CA, USA) for 20 min at room temperature. After washing the cells three times with 3% BSA in PBS, a 100 μL 1 × Click-iT^®^ reaction cocktail was added into each well, and the plate was incubated for 30 min at room temperature in the dark. Then, 1 ml of 1× Hoechst 33342 nuclear staining solution (Sigma, San Francisco, CA, USA) was added into each well, and the plate was incubated for 25 min at room temperature in the dark. Subsequently, the staining solution was removed, and the cells were washed three times with PBS. Then, the EdU-labeled cells were photographed and counted using a fluorescence microscope (CKX41-F32FL, Olympus, Tokyo, Japan). Image-Pro Plus software (Version 5.0, MD, USA) was used to determine the percentage of EdU-positive (EdU+) cells.

**Colony formation assay**

3 ml complete medium with 560 cells per well was seeded in 6-well plates. The cells were cultured at 37°C and 5% CO_2_ for two weeks and the medium was not changed during this period. Then, the cells per well were washed with PBS three times and fixed with 4% formaldehyde. The colonies were stained with 0.1% crystal violet (Servicebio, Wuhan, China). At the end, the colonies with a diameter > 2 mm were photographed and counted under an inverted microscope. All the experiments were repeated three times independently.

**Cell migration and invasion assays**

Cell migration and invasion experiments were performed using the Transwell system (Corning, NY) based on the manufacturer’s instructions. To assess invasion, filters were precoated with Matrigel (BD Biosciences, San Josè, CA, USA). Approximately 3 × 10^5^ cells were added into the top chamber containing serum-free DMEM. The bottom chamber contained 600 microliters of DMEM supplemented with 20% fetal bovine serum (FBS). Following 24 h of incubation, the cells on the upper surface were gently removed with a cotton swab, and then the membrane was fixed with 4% formaldehyde for 20 min and stained with 0.1% crystal violet solution (Servicebio, Wuhan, China) for 30 min. The cells that migrated to the lower surface of the membrane were photographed and counted under a microscope. The same experiment was performed for the migration assays except that the filters were not precoated with Matrigel.

**Dual luciferase reporter assay**

U-87MG and U-138MG cells were routinely plated in 24-well plates for 24 h before transfection. The cells were transfected with JAK/STAT firefly luciferase reporter plasmid and phRL-TK (Origene, Rockville, MD, USA) using Lipofectamine™ 3000 (Invitrogen, CA, USA) according to the manufacturer’s protocol. The Renilla luciferase expression plasmid acted as an internal control. Then, the cells were harvested 24 h posttransfection and lysed with 100 μl of 1 × passive lysis buffer (Boster, Wuhan, China). Subsequently, the luciferase activities were calculated with a Dual-Glo Luciferase kit (Promega, USA).

**Cignal finder cancer 10-pathway reporter array**

Pathway analyses were performed with the Cignal Finder Cancer 10-Pathway Reporter Array (QIAGEN, Germany) according to the manufacturer’s instructions. The suspended cells (8 × 10^4^/ml, 60 μl/well) were seeded into 96-well plates containing the luciferase reporters to target common cancer pathways. Then, the cells were incubated at 37°C and 5% CO_2_ for 24 h, and luciferase activity analyses were performed using the Dual-Luciferase Reporter Kit (Promega, USA).

**Western blotting**

The cells were lysed by electrophoresis on a 12% SDS-PAGE gel at 120 V for 2 h and were then transferred to a PVDF membrane (Millipore, Massachusetts, USA). The membrane was blocked with 5% fat-free milk in PBS for 2 h. Next, the membranes were incubated with primary and secondary antibodies and visualized using a chemiluminescent reagent (Thermos).

**qRT-PCR**

Total RNA was lysed from glioma samples using TRIZOL reagent (Invitrogen). cDNA synthesis and real-time PCR were performed using the SYBR® Premix Ex Taq™ Kit (Takara, Japan). GAPDH was acted as an internal control. The sequences of the ELTD1 primers were as follows: forward, 5’-CAGACAGAACTACTAACTTCGCC-3’; reverse, 5’-ATGCCTCGGATGAAGAGTCCT-3’. The sequences of the HIF-1α primers were as follows: forward, 5’-AGCCTGTGGAAAGACATGCTT-3’; reverse, 5’-TCAAACACTGTGGGCACATAC-3’. Relative mRNA expression levels were normalized as described previously.

**Co-IP (Coimmunoprecipitation)**

Infected U-87MG and U-138MG cells were lysed in immunoprecipitation (IP) buffer. The cell lysates were centrifuged at 10,000 g for 25 min at 4°C and then incubated with anti-p-STAT3 or anti-HIF-1α antibody covalently coupled to protein A/G-agarose beads (Servicebio, Wuhan, China) overnight on a spinning wheel at 4°C. The parallel IgG antibody acted as a negative control. Prior to the addition of antibodies, a certain proportion of each supernatant without any antibody (Input) was used as a positive control. The pellets were washed three times with IP buffer. The collected proteins were eluted from the beads by boiling them in 12% SDS-PAGE loading buffer (Boster, Wuhan, China) for 5 min, and the proteins were then subjected to Western blotting.

**Immunofluorescence and immunohistochemical staining**

Immunofluorescence (IF) staining was performed as described previously. Cells were seeded in 24-well plates and fixed with 4% formaldehyde. After fixation, the cells were permeabilized with 0.5% Triton X-100 and blocked with 5% bovine serum albumin (BSA) in PBS for 1 h at room temperature. Then, the cells were incubated with anti-ELTD1 (1:300) antibody in 0.3% BSA in PBS and were finally incubated with a Cy3-conjugated secondary antibody (1:100, Promoter, China) in 0.3% BSA in PBS. Fluorescence was captured using a Laser Scanning Confocal Microscope (Olympus, Japan).

Immunohistochemistry and semiquantitative scoring techniques were performed as described previously. The percentage of positive staining was defined as follows: 0 (no positive), 1 (0–10%), 2 (10–30%), 3 (30–70%), and 4 (70–100%). The staining intensity was graded as follows: 1 (no staining), 2 (weak staining), 3 (moderate staining), and 4 (strong staining). The staining index (SI) was multiplied with possible scores of 0, 1, 2, 3, 4, 6, 8, 9, 12, and 16, and the median value was SI = 8, which was chosen as the cut off value. Therefore, samples with SI ≥ 8 had high expression, and samples with SI < 8 had low expression. IHC analyses were independently performed by two experienced pathologists who were blinded to the tissue information to avoid evaluation biases.

**Brain orthotopic xenografts**

All animal experiments were approved by the Institutional Animal Care and Use Committee of Tongji Medical College, Huazhong University of Science and Technology (S841). Nude mice (8 weeks old) were purchased from Beijing Vital River Animal Technology Co., Ltd. (Beijing, China), and five mice were allocated to each group. The head of the mouse was fixed using a stereotactic apparatus, and the skull over the right hemisphere of the brain was exposed via a skin incision to inject into the brain. The skull was drilled using a high-speed air-turbine drill (a burr tip size of 0.5 mm in diameter) until a bone flap became loose. Approximately 5 μL of U-87MG-Luc or U-138MG-Luc (ELTD1/HIF-1α-luciferase, vector-luciferase, shELTD1-luciferase or shControl-luciferase) cell suspension (10 × 10^7^ cells/ml in PBS) that stably expressed firefly luciferase was injected into the brain parenchyma using a microliter syringe. Then, the bone flap was placed back and sealed with histocompatible cyanoacrylate glue. Subsequently, the skin on the skull was sutured closed. Tumors were monitored and quantified using a bioluminescence imaging system (PerkinElmer, IVIS Spectrum Imaging System, USA) after four weeks. For bioluminescence imaging, mice received D-Luciferin Firefly, sodium salt (10 ul/g, 15 mg/ml, Glod Biotechnology, Inc., 10 min before imaging) and were anesthetized with 1% pentobarbital, followed by imaging in an IVIS spectrum imaging system.


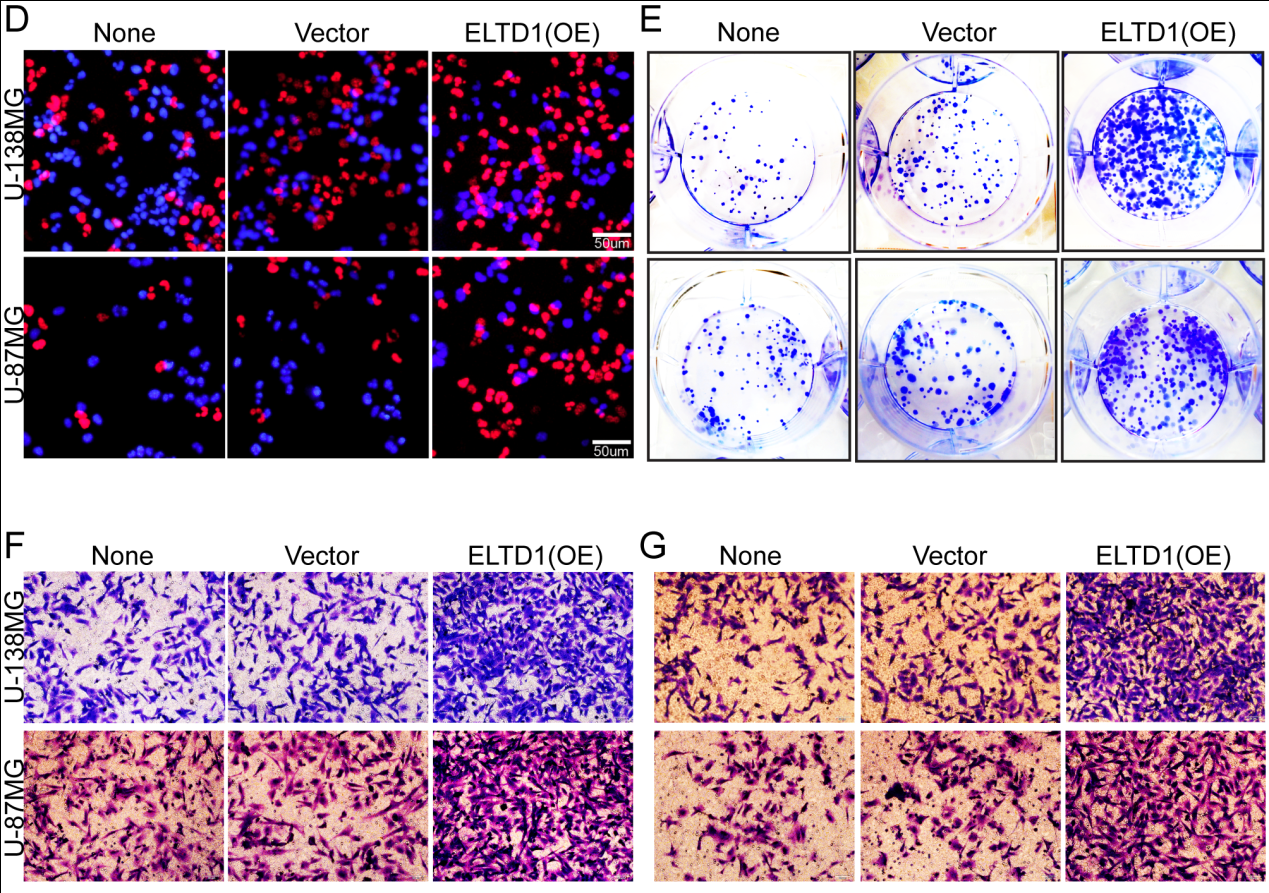


Supplement 1: Representative images of the EdU (D), colony formation (E), Transwell migration (F) and invasion assays (G) with U-138MG and U-87MG cells.


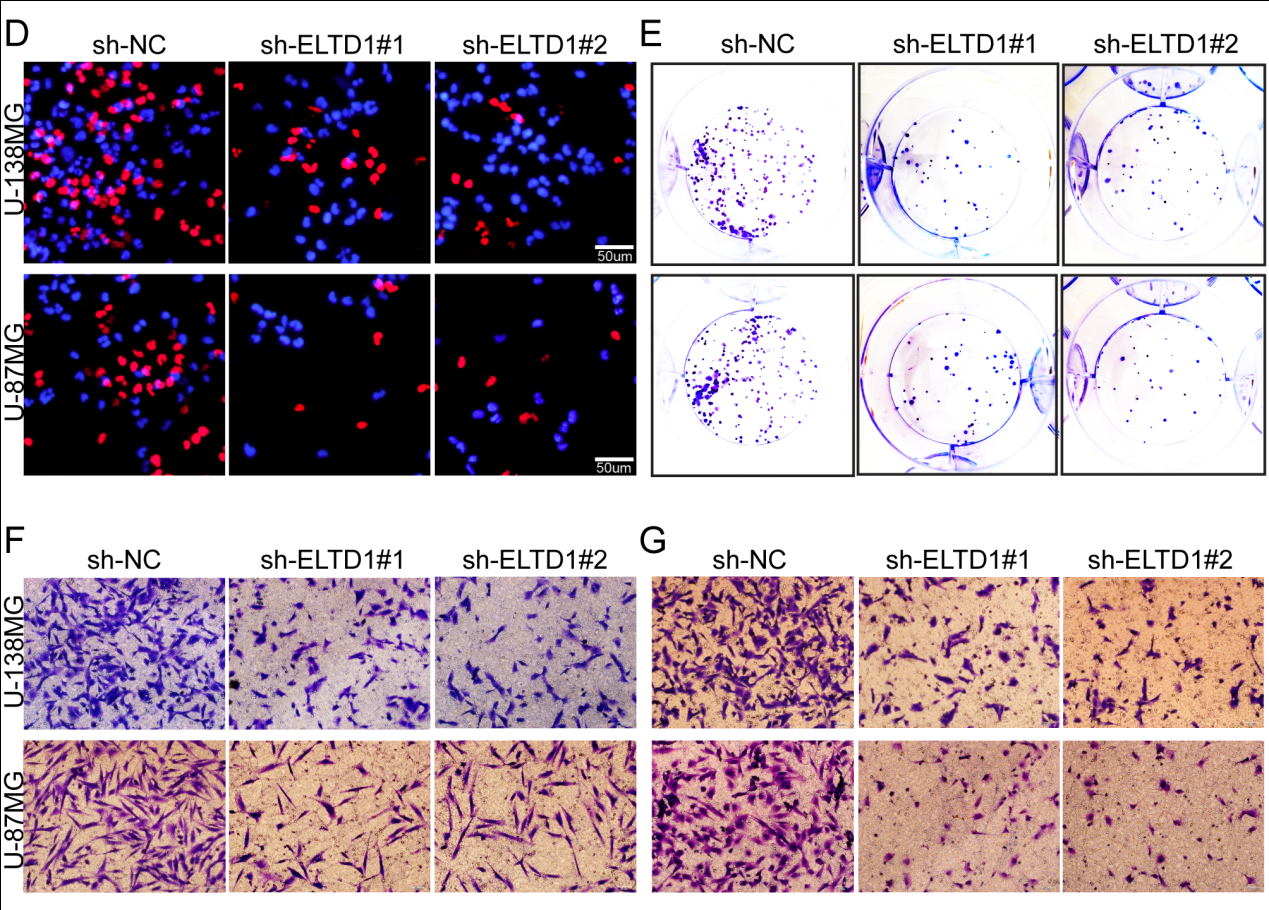


Supplement 2: Representative images of the EdU (D), colony formation (E), Transwell migration (F) and invasion assays (G) with U-138MG and U-87MG cells.


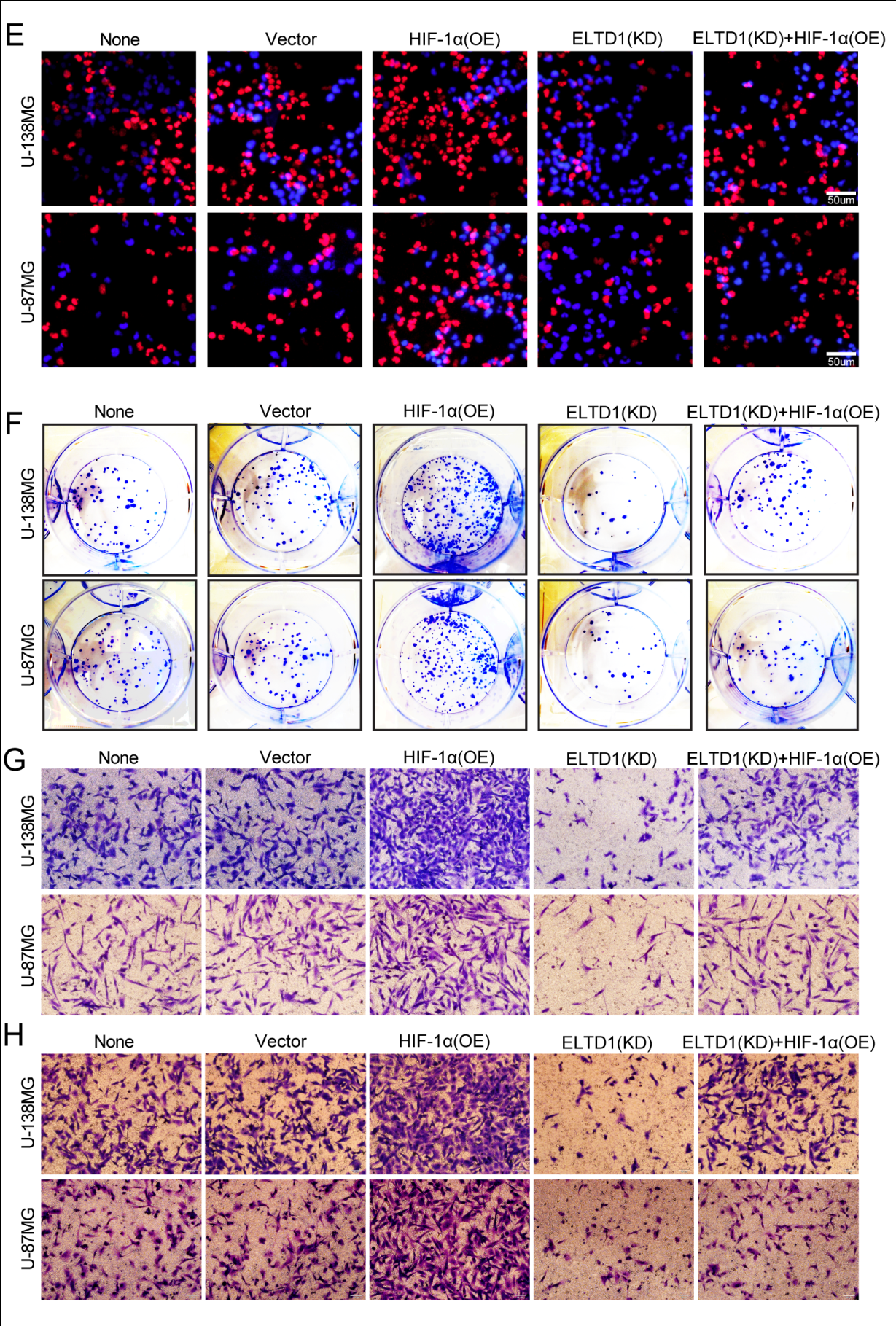


Supplement 3: Representative images of the EdU (E), colony formation (F), Transwell migration (G) and invasion assays (H) with U-138MG and U-87MG cells.


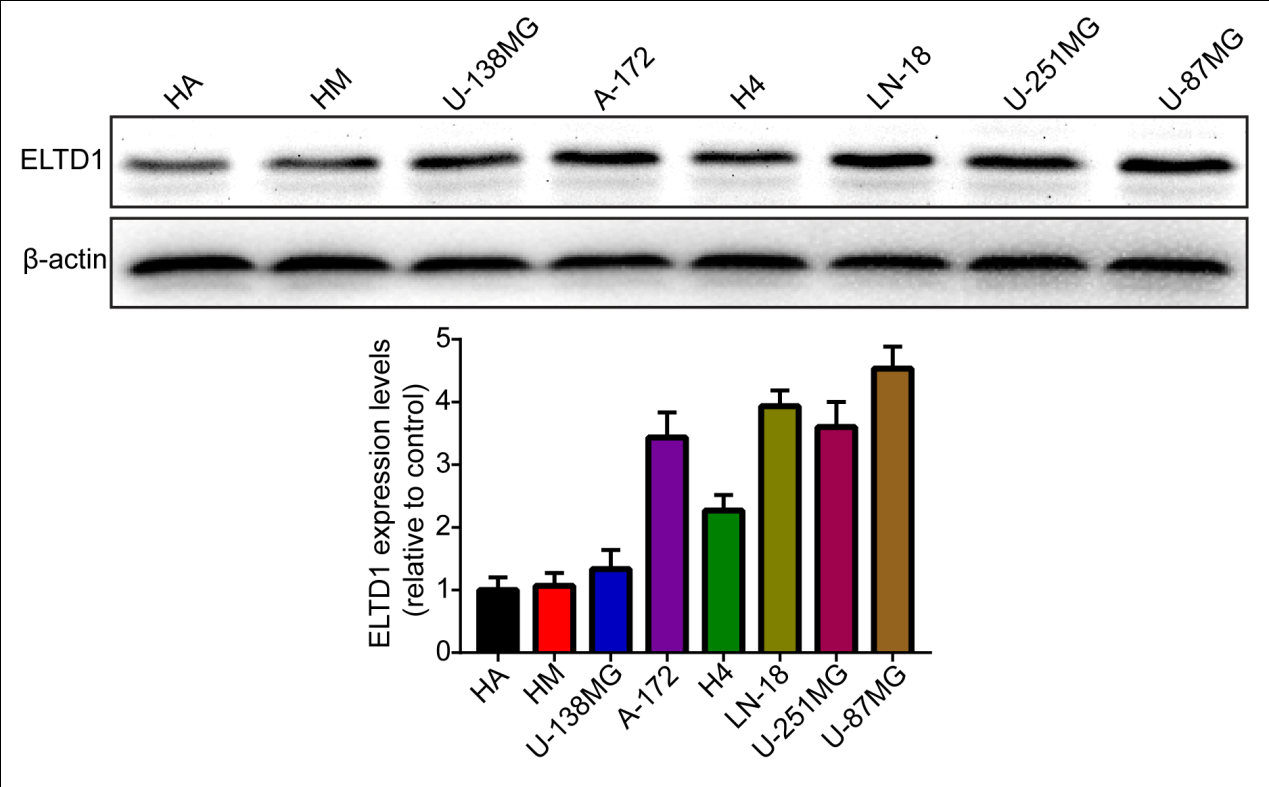


Supplement 4: Relative ELTD1 expression levels measured by Western blotting and RT-PCR in two human brain gliocyte cell lines (HA and HM) and six human brain glioma cell lines (H4, A-172, U-138MG, LN-18, U-87MG and U-87MG).


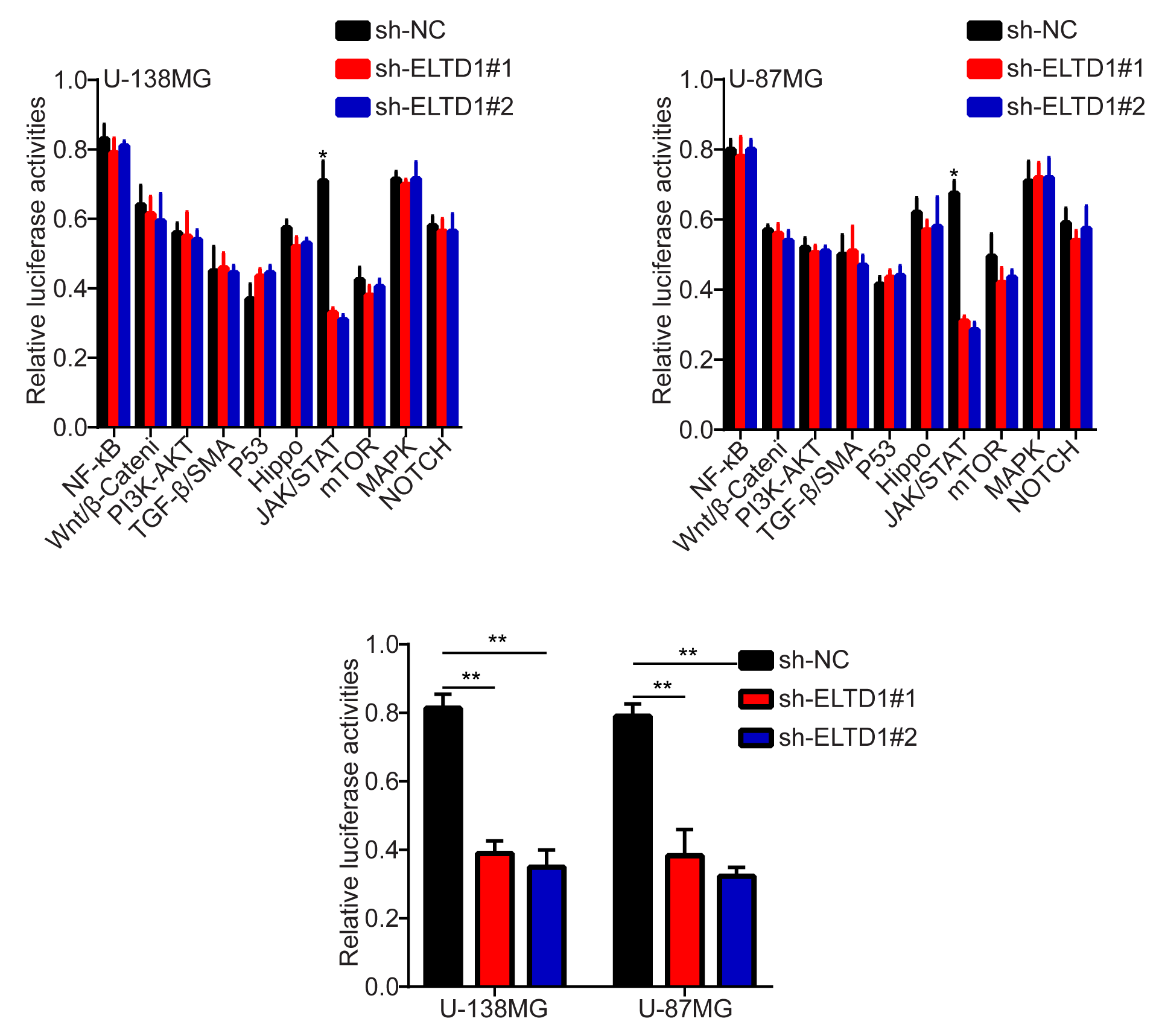


Supplement 5: ELTD1 knockdown decreased the luciferase activities of JAK/STAT by the dual luciferase reporter assay. Others remain unchanged. **p < 0.01.


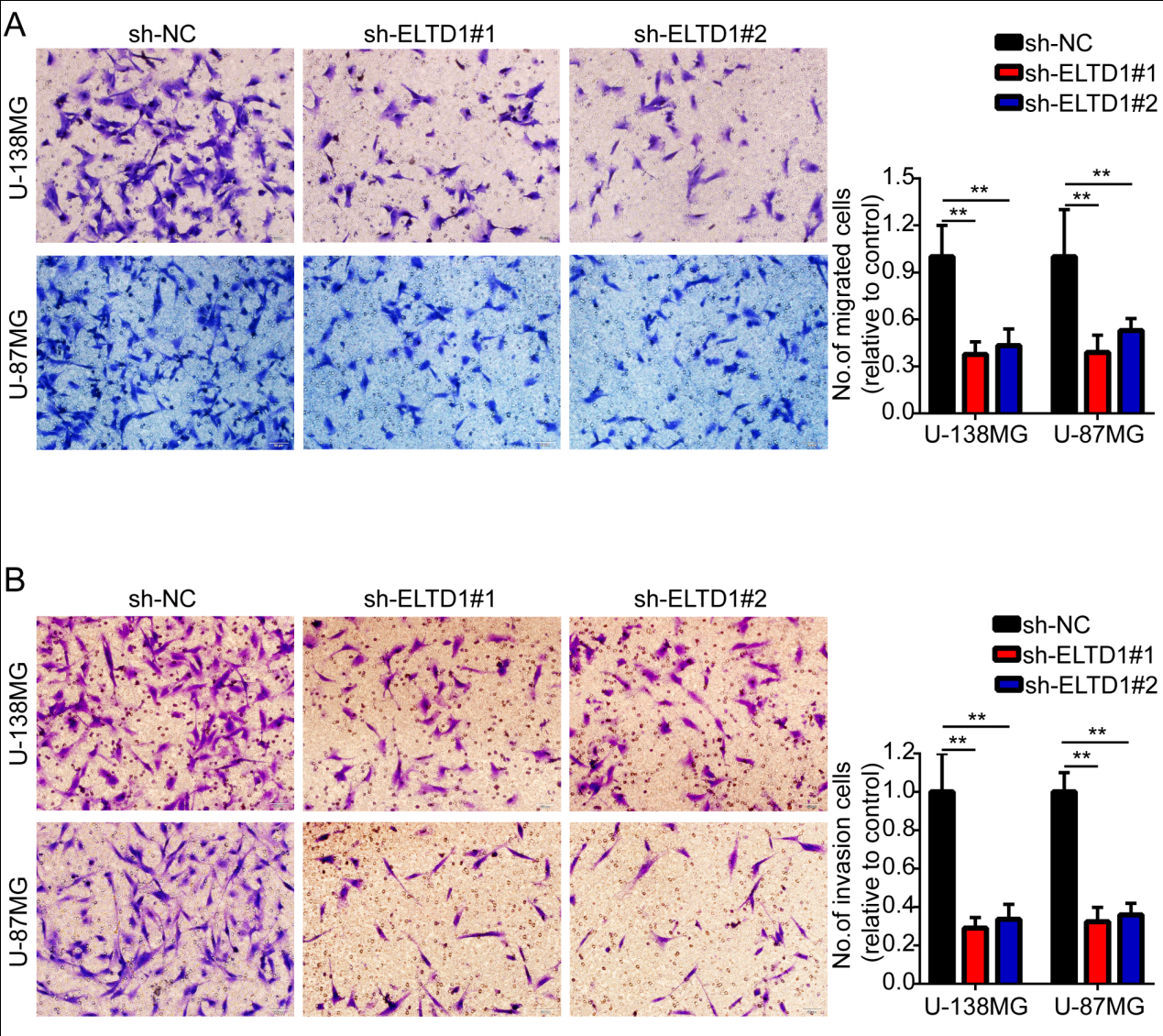


Supplement 6: Representative images (left panels) and histogram quantification (right panels) of the Transwell migration (A) and invasion assays (B) with U-138MG and U-87MG cells. Statistical significance was assessed using one-way ANOVA followed by Dunnett’s tests for multiple comparisons. Scale bars: 50 μm. **p < 0.01.


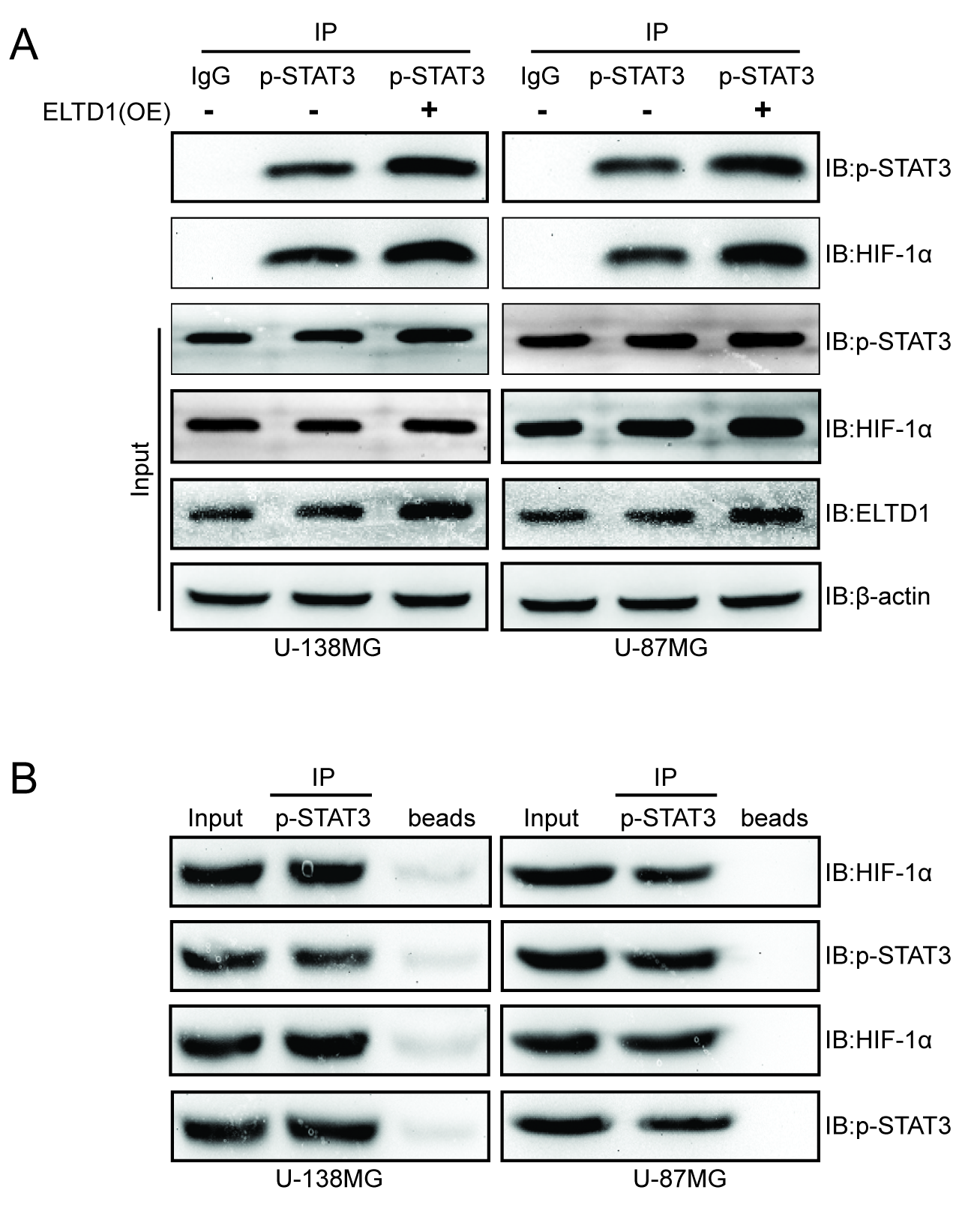


Supplement 7: Co-IP experiment indicated that p-STAT3 could bind with HIF-1α.


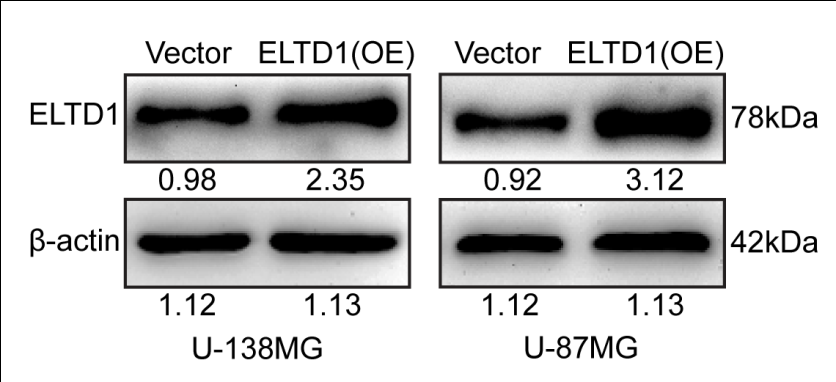


Supplement F2-B: The overexpression efficiency against ELTD1 was verified by Western blotting in U-138MG and U-87MG cells.


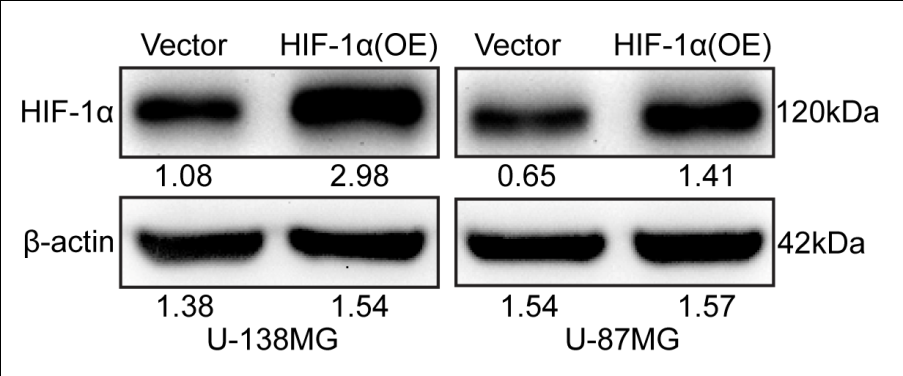


Supplement F5-C: The overexpression efficiency against HIF-1α was verified by Western blotting in U-138MG and U-87MG cells.
