# Supplementary material for: ELTD1 facilitates glioma proliferation, migration and invasion by activating JAK/STAT3/HIF-1α signaling axis
Source: Sci Rep. 2019 Sep 25;9:13904. doi: 10.1038/s41598-019-50375-x (PMC6761139; doi:10.1038/s41598-019-50375-x)
Supplement: Supplementary file 2 — Language Certificate [file 41598_2019_50375_MOESM2_ESM.pdf]

AMERICAN JOURNAL EXPERTS

EDITORIAL CERTIFICATE

This document certifies that the manuscript listed below was edited for proper English language, grammar, punctuation, spelling, and overall style by one or more of the highly qualified native English speaking editors at American Journal Experts.

Manuscript title:

ELTD1 facilitates glioma proliferation, migration and invasion by activating JAK/STAT3/HIF-1α signaling axis

Authors:

Junjun Li1 , Hao Xu1 , Qiangping Wang1 , Songshan Chai1, Peng Fu 1, Lei Wang1 , Hongyang Zhao1, Jinsong Li2, Nanxiang Xiong1

Date Issued:

January 2, 2019

Certificate Verification Key:

19EB-8279-99BC-5D7F-C131

This certificate may be verified at www.aje.com/certificate. This document certifies that the manuscript listed above was edited for proper English language, grammar, punctuation, spelling, and overall style by one or more of the highly qualified native English speaking editors at American Journal Experts. Neither the research content nor the authors' intentions were altered in any way during the editing process. Documents receiving this certification should be English-ready for publication; however, the author has the ability to accept or reject our suggestions and changes. To verify the final AJE edited version, please visit our verification page. If you have any questions or concerns about this edited document, please contact American Journal Experts at support@aje.com.

American Journal Experts provides a range of editing, translation and manuscript services for researchers and publishers around the world. Our top-quality PhD editors are all native English speakers from America's top universities. Our editors come from nearly every research field and possess the highest qualifications to edit research manuscripts written by non-native English speakers. For more information about our company, services and partner discounts, please visit www.aje.com.
